# “AH, A PROGRAM FOR FORMER CAREGIVERS. WHAT A GREAT IDEA! WHY DON’T YOU START SOMETHING?”

**DOI:** 10.1093/geroni/igad104.0031

**Published:** 2023-12-21

**Authors:** Sheryl Fairbanks, Warren Wolfe, Ashley Millenbah, Robbin Frazier, Zachary Baker

**Affiliations:** University of Minnesota, Minneapolis, Minnesota, United States; Freelance Journalist, Roseville, Minnesota, United States; University of Minnesota, Minneapolis, Minnesota, United States; University of Minnesota, Center for Healthy Aging and Innovation, Minneapolis, Minnesota, United States; Arizona State University, Tempe, Arizona, United States

## Abstract

Sheryl Fairbanks and Warren Wolfe had insight into the experiences of bereaved dementia caregivers, having cared for four parents, two of whom had dementia, who all died within 14 months of each other after 12 years of care. After taking some time to grieve and settling an entire generation’s estates, Ms. Fairbanks and Mr. Wolfe sought resources to help them re-engage in life following caregiving. Here they were uniquely qualified to find what resources might exist. Mr. Wolfe had been a newspaper reporter and editor for four decades. Two of these decades were spent covering aging and in 2010 he was a member of the GSA Journalists in Aging Fellows Program. Likewise, Ms. Fairbanks attended GSA in 2015 as a journalist and editor as they contacted experts across the country seeking resources for bereaved dementia caregivers. While many sympathized with the need for such resources, few could be found, and it was suggested that maybe Mr. Wolfe and Ms. Fairbanks ought to create a group to help bereaved dementia caregivers figure out what was next after caregiving. In 2016 they created the Former Dementia Caregiver Re-Entry Initiative, which has helped more than 60 members figure out the next steps for themselves, re-engaging with life after caregiving. They have talked about hundreds of topics, laughed a lot, cried at times, disclosed deep fears, shared small and big discoveries about themselves, and generally have found a firmer footing as they have embarked on this new journey.

